# Risk score prediction model based on single nucleotide polymorphism for predicting malaria: a machine learning approach

**DOI:** 10.1186/s12859-022-04870-0

**Published:** 2022-08-07

**Authors:** Kah Yee Tai, Jasbir Dhaliwal, KokSheik Wong

**Affiliations:** School of Information Technology, Monash University Malaysia, Subang Jaya, Selangor Malaysia

**Keywords:** Malaria, Single nucleotide polymorphisms, Machine learning, Feature extraction algorithm, Genetic risk factors, Weighted genetic risk score

## Abstract

**Background:**

The malaria risk prediction is currently limited to using advanced statistical methods, such as time series and cluster analysis on epidemiological data. Nevertheless, machine learning models have been explored to study the complexity of malaria through blood smear images and environmental data. However, to the best of our knowledge, no study analyses the contribution of Single Nucleotide Polymorphisms (SNPs) to malaria using a machine learning model. More specifically, this study aims to quantify an individual's susceptibility to the development of malaria by using risk scores obtained from the cumulative effects of SNPs, known as weighted genetic risk scores (wGRS).

**Results:**

We proposed an SNP-based feature extraction algorithm that incorporates the susceptibility information of an individual to malaria to generate the feature set. However, it can become computationally expensive for a machine learning model to learn from many SNPs. Therefore, we reduced the feature set by employing the Logistic Regression and Recursive Feature Elimination (LR-RFE) method to select SNPs that improve the efficacy of our model. Next, we calculated the wGRS of the selected feature set, which is used as the model's target variables. Moreover, to compare the performance of the wGRS-only model, we calculated and evaluated the combination of wGRS with genotype frequency (wGRS + GF). Finally, Light Gradient Boosting Machine (LightGBM), eXtreme Gradient Boosting (XGBoost), and Ridge regression algorithms are utilized to establish the machine learning models for malaria risk prediction.

**Conclusions:**

Our proposed approach identified SNP *rs334* as the most contributing feature with an importance score of 6.224 compared to the baseline, with an importance score of 1.1314. This is an important result as prior studies have proven that *rs334* is a major genetic risk factor for malaria. The analysis and comparison of the three machine learning models demonstrated that LightGBM achieves the highest model performance with a Mean Absolute Error (MAE) score of 0.0373. Furthermore, based on wGRS + GF, all models performed significantly better than wGRS alone, in which LightGBM obtained the best performance (0.0033 MAE score).

**Supplementary Information:**

The online version contains supplementary material available at 10.1186/s12859-022-04870-0.

## Background

Malaria is a mosquito-borne infectious disease that can progressively infect and annihilate the red blood cells. The World Health Organization’s report entitled “World Malaria Report” [1] estimated the annual death of 400,000 from malaria, of which two-thirds are children below the age of five. It is also more commonly diagnosed in sub-Saharan Africa, where a significantly large number of cases throughout the endemic region are due to increased mosquitoes' reproduction resulting in more malaria transmission. Furthermore, ineffective prevention methods are still widespread [2]. Thus, much research has been devoted to developing tools for malaria surveillance. It is believed that prevention is better than cure [3], and hence, the implementations of preventative solutions are crucial in determining individuals’ early detection even before the disease symptoms strike.

The development of malaria cases in malaria-endemic regions is not the only known risk factor. According to other observations in the literature [4–6], specific inherited genetic traits are also possible risks of malaria contraction among different populations. Thus, genetic susceptibility (an inherited increase in the risk of developing the disease) [7] and genetic resistance (the ability to prevent the risk of developing the disease or a marked reduction in the severity of the symptoms) to malaria have been characterized by genetic mutations with erythrocytes. This includes hemoglobin variants or related sickle cell disease, Duffy blood antigens glycophorins, and blood type groupings. Substantial evidence in the past decade has established that different populations have different susceptibility to malaria due to diverse genetic adaptations and gene selection pressures of malaria in the human genome [8].

A genome-wide association study (GWAS) is an approach used in genetics research that scans genomes of multiple individuals to find specific genetic variations with a particular disease. These genetic variations are markers or contributing risk factors of the disease [9]. Multiple prior studies [10, 11] have established that the Hemoglobin Subunit Beta (*HBB*) gene to be a significant genetic risk factor for malaria because of its risk alleles. However, *HBB* variants alone cannot precisely predict the disease as malaria is a complex disease. Many other genes, such as *ABO*, *ATP2B4*, *G6PD*, *CD40LG, FY, GYPA, GYPB, GYPC, HBA, HP, SCL4A1,* have been associated with malaria susceptibility or resistance across different populations via GWAS [12]. To further combat the disease's spread, it is crucial to understand the contributing genetic risk factors involved in characterizing the disease's complexity [10].

Machine learning is an Artificial Intelligence strategy that uses training algorithms to learn from large datasets, and is the basis of numerous developments, from speech recognition to autonomous self-driving cars [13]. Inspired by the recent breakthroughs of machine learning in various domains, this paper applies machine learning algorithms to study malaria’s complexity by leveraging well-planned malaria research on genetic variants. The Malaria Genomic Epidemiology Network (MalariaGEN) http://www.malariagen.net/ is a community of researchers from more than 20 countries to understand how natural genetic variation in humans and malaria parasites affects the biology and epidemiology of malaria. This network, formally established in 2005, has developed several GWAS [12, 14–22] with their collaborating partners to fulfil their goals. Therefore, MalariaGEN produces the essential data required for machine learning algorithms to mine for valuable patterns.

In recent endeavours, machine learning algorithms were used to explore the complexity of malaria, particularly malaria parasites and development stages, through blood smear images [23, 24]. In separate studies, environmental data were collected and trained by machine learning algorithms to link climate change to malaria transmission [25, 26]. Since the aforementioned prediction strategy was deemed successful, we leveraged the power of machine learning to quantify the risk score of an individual’s susceptibility to malaria based on genetic variation, i.e., the contributing risk factors, instead. Currently, the prediction is limited to using advanced statistical methods, such as time series and cluster analysis [27]. Hence, machine learning prediction models based on genetic variation are required to fully explore the disease's potential genetic markers. The machine learning algorithms can detect valuable patterns in complex datasets by applying various optimization, statistical and probabilistic methods to identify the most suitable set of variables to train the model, for example, Single Nucleotide Polymorphisms (SNPs). SNP is the most common type of genetic variation among individuals. We are interested in finding out whether it is possible to quantify an individual’s risk of malaria based on SNP genotype data for facilitating personalized prevention and treatment. Thus, instead of classifying “Is this individual infected by malaria?”, this study predicts a risk score to answer the question “What is the individual risk score towards malaria susceptibility?”. In answering this question, we propose a feature extraction algorithm that aids in selecting the main SNP features representing the genetic risk factors as well as exploring machine learning models’ performance based on SNP genotype data. Weighted genetic risk scores (wGRS) and the combination of wGRS with genotype frequency (wGRS + GF) were calculated as the target variable. The contributions of this paper are summarized below:

- Proposes a novel feature extraction algorithm using genotype patterns that can aid Logistic Regression and Recursive Feature Elimination (LR-RFE) in selecting the significant features. We also provide the ranked features list obtained from our approach;
- Proposes a novel machine learning-based model to predict the risk score of an individual developing malaria, and;
- Proposes a risk score that combines wGRS and genotype frequency (wGRS + GF). We also provide a comprehensive analysis of the experimental results, comparing wGRS with wGRS + GF.

This study hypothesizes that we can predict the risk score of an individual’s susceptibility to malaria from genetic variants through the proposed feature extraction algorithm with LR-RFE and the analysis of multiple machine learning models. The potential findings will add to our understanding of this topic by exploring genetic variation for different populations to predict an individual’s risk score of developing malaria, i.e., individual malaria risk score, in various regions. This will further create a comprehensive understanding of malaria susceptibility and reaching out to a wider community to combat the spread of malaria.

## Methods

**Fig. 1:**
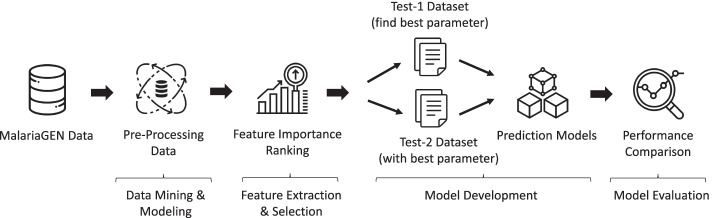
Machine learning pipeline for individual malaria risk score prediction

**Fig. 2:**
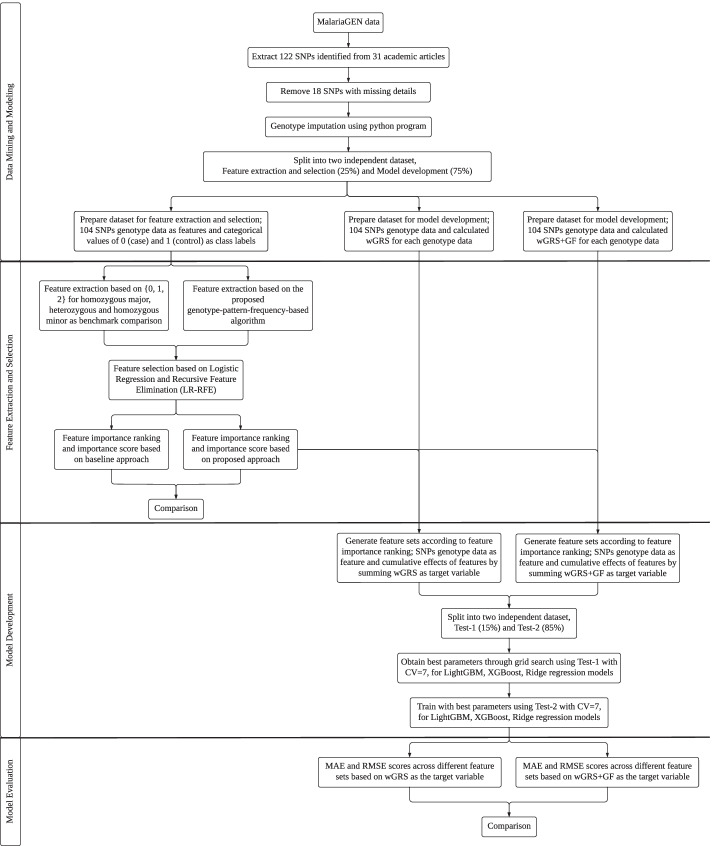
Methodology flow chart

Figure 1 depicts the pipeline for identifying the malaria risk score in subjects, starting from the raw data until the evaluation stage of the prediction model. The pipeline consists of four discrete stages of operation: (1) Data Mining and Modeling, (2) Feature Extraction and Selection, (3) Model Development and (4) Model Evaluation. Figure 2 shows the methodology flow chart in detail.

### Dataset

Our study uses the human GWAS data produced from the MalariaGEN Consortial Project 1 entitled: “Genome-wide study of resistance to severe malaria in eleven populations.” The consortial project structure is described in [28], and information on each collaborating partner's studies and field sites is acknowledged on the MalariaGEN website.

We used genotype data of 20,854 individuals (10,791 malaria-affected individuals and 10,063 controls) from 11 worldwide populations (Table 1).

**Table 1:** Analysed populations and samples

Population	Case	Control	Sample size
Burkina Faso	807	639	1446
Cameroon	693	778	1471
Gambia	2807	2786	5593
Ghana	422	342	764
Kenya	1944	1738	3682
Malawi	1590	1498	3088
Mali	475	394	869
Nigeria	288	131	419
Tanzania	485	494	979
Vietnam	860	868	1728
Papua New Guinea	420	395	815
Total			20,854

Sample size indicates the total number of individuals for each population

### Data mining and modeling

#### Data preprocessing

Initially, the first stage of the pipeline is converting raw MalariaGEN genotype data into a format that machine learning models can use. We downloaded the data from the European Genome-phenome Archive http://ega-archive.org/. We then identify 122 SNPs relevant to malaria through literature reviews, where 31 academic articles [8, 12, 15–22, 29–49] are reviewed and analysed. Out of 122 SNPs, 18 SNPs are removed due to unreported effect size and unavailability in some populations. A total of 104 SNPs is retained (Additional file 1), with genotypes comprising major allele *A* and minor allele *a*. This stage also converts all unparseable values in the data, such as data types and standard format errors, into null representations. We also map 32 kgpIDs to rsIDs and remove 37 samples without detailed information about malaria subtypes. This preprocessing procedure yielded 104 SNP variables from a total of 20,817 samples.

Generally, genotype imputation softwares such as IMPUTE2 [50] and Beagle [51] are deployed for estimating missing genotypes. These software programs impute missing genotypes based on publicly available reference datasets such as 1000 Genomes Project or HapMap 3. However, in our case, imputation needs to be more specific as we are developing a prediction model of individual’s susceptibilities to malaria. Thus, we developed a python program that imputes any missing genotypes based on the population group and malaria subtype from the human GWAS data used in this study. In order to do so, the program first groups individuals based on their countries and then by their malaria subtypes. Finally, a comparison of a total of six SNPs for each missing genotype, i.e., three SNPs before and after the missing loci, is performed before imputing the missing genotype with the most common genotype data.

Moreover, to perform feature extraction and selection separately from model development, we split the preprocessed dataset into two independent datasets, of which 25% were used for feature extraction and selection (5,204 samples; 2,667 malaria-affected individuals and 2,537 controls), and 75% were used for model development (15,613 samples; 8,087 malaria-affected individuals and 7,526 controls).

#### Dataset for feature extraction and selection

As a preparation for the feature extraction and selection stage, we represent the genotype data as feature and class label data frame. Each SNP itself is a feature, where the columns represent 104 SNPs containing genotype data. The genotype data is formed by two alleles, *A* and *a*, generally expressed as *AA*, *Aa,* and *aa*. The last column representing the class label contains the categorical value representing the binary classification of the individuals: 0–case (malaria-affected) and 1–control (healthy). Note that binary classification is only used for feature extraction and selection; for model development, the target variable is the risk score described in the next section.

#### Dataset for model development

The model is designed to provide individuals with continuous risk scores rather than binary classification. Therefore, we convert the categorical value in the class label by calculating wGRS as the target variable. To further evaluate the model performance of wGRS, we also computed wGRS + GF as the target variable for an identical dataset, which will be compared with wGRS-only model to determine the better performing model.

#### Genotype patterns and frequency

Several observations in the literature [52, 53] indicate that genotype patterns contribute to disease association. The exploration of genotype patterns is significant for malaria prediction as substantial evidence has established that sickle cell anemia traits can partially prevent malaria [10, 54–56]. The genetic trait of sickle cell anemia is found on the recessive allele of the hemoglobin gene. This means that an individual needs to have two copies of the recessive alleles—one from the mother and one from the father—to have this condition. If the alleles are heterozygous, the individual tends to be resistant to the development of malaria. In contrast, if the alleles are homozygous, the individual is susceptible to the development of malaria. Inspired by these evidences, our study will include genotype frequency in the formulation of wGRS + GF to characterize the individual’s malaria risk score; and propose a novel feature extraction algorithm based on genotype patterns and frequencies.

#### wGRS

The wGRS is calculated for each genotype by multiplying the number of risk alleles (0, 1, 2) by the estimated effect size reported for each variant. The reported effect size was estimated using the logistic regression association tests method and can be found in the association test summary statistics on the MalariaGEN website https://www.malariagen.net/sppl25/. The calculation of wGRS is summarized as: *risk allele* * *effect size* = wGRS [57].

#### wGRS + GF

The wGRS + GF combines the wGRS mentioned above and genotype frequency. Similarly, the wGRS + GF is calculated for each genotype. The Hardy–Weinberg equation is utilized to calculate genotype frequency from genotype data as this equation calculates an individual’s genetic variation at equilibrium. For example, the frequency of allele *A* is represented by *p*, and the frequency of allele *a* is represented by *q*. Thus, the frequency of genotype *AA* is calculated as *p*^*2*^, genotype *aa* is calculated as *q*^*2*^, and genotype *Aa* is calculated as *2pq*. The wGRS + GF is then produced by multiplying the genotype frequency by the wGRS. The calculation of wGRS + GF is summarized as: (*risk allele* * *effect size*) * *genotype frequency* = wGRS + GF*.* Of note, this approach is a novelty, because researchers to date only used wGRS [58–60]. However, the wGRS only considers the risk alleles and the variant effect size, which is insufficient in these aspects for malaria prediction as genotype patterns have been associated with malaria. Thus, it is essential to include genotype frequency to characterize an individual’s malaria risk score further.

### Feature extraction and selection

#### Feature extraction

We proposed an algorithm to extract genotype, i.e., SNP features, into a valuable set of information that aids feature selection. As mentioned earlier, for all subjects, genotype data is usually represented as *AA, Aa,* and *aa*, by two alleles *A* (major allele) and *a* (minor allele). Such representation leads to repeated data that may affect analysis performance, and thus feature extraction and normalization are required. The proposed algorithm focuses on genotype patterns where we find pattern frequencies based on populations due to its strong linkage between genotype patterns and malaria. This approach is a novelty because the current state-of-the-art methods substitute genotype data with numerical values {0, 1} for the major and minor allele [61]; or with numerical values {0, 1, 2} for homozygous major, heterozygous and homozygous minor [62–65]. Simultaneously, some other studies rely on odd ratios calculated from genotype data [60], and some are combined with clinical features [66].

**Fig. 3:**
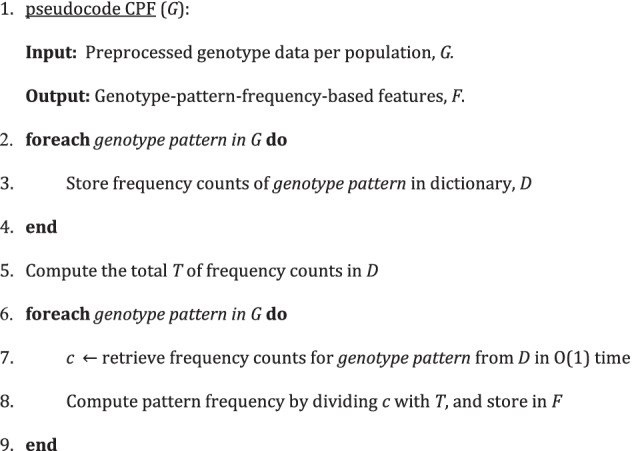
Pseudocode of the proposed feature extraction algorithm

For each population, the proposed feature extraction algorithm makes a left-to-right pass over the preprocessed dataset to compute the frequency counts of the genotype patterns. The genotype patterns and frequency counts are stored in a dictionary. Next, we compute the total of all the genotype patterns by summing up all the counts in the dictionary. The total is used for data normalization. Another left-to-right pass is then made over each population's preprocessed dataset, but this time, we retrieve the genotype pattern counts from the dictionary in O(1) time to divide it by the total, which we define the resulting value as the pattern frequency. Each pattern frequency is stored based on the SNP feature list. The pseudocode of the proposed feature extraction algorithm is presented in Fig. 3. The most common feature extraction approach that substitutes homozygous major, heterozygous and homozygous minor with numerical values {0, 1, 2} is used for benchmark comparisons.

#### Feature selection

To create an accurate model that relies on the most relevant features (namely, features that contribute the most for malaria prediction), we evaluated the feature importance, i.e., feature dependence of the model, through Logistic Regression and Recursive Feature Elimination (LR-RFE). The analysis is based on the feature extraction algorithm described above, followed by the Recursive Feature Elimination (RFE) algorithm, in which the Logistic Regression (LR) coefficient is utilized in the core of the model to perform feature selection.

The LR model’s coefficients have been widely utilized for feature importance estimation [67]. Each coefficient represents a score, known as the feature importance score, which describes the significance level between the feature and the target variable. The higher the coefficient, the more relevant the feature is to the target variable. In other words, coefficients can be utilized to determine the important and unimportant features to avoid overfitting [67] and are thus useful for prediction [68]. The RFE model ranks the 104 features based on their importance scores obtained from the LR model into a list, in which the first position represents the most significant feature, while the least important feature is ranked on the last position. The features are ranked via their importance scores, where the least important features are iteratively eliminated through remodeling until the required number of features is retained. This iterative process results in a list of ranked features, i.e., from the most to the least important features.

In this regard, a recent study [69] used LR-RFE to rank feature importance and selection to find the optimal feature set in breast cancer prediction. The study focused on cytological characteristics obtained from the breast fine needle aspiration test. Several models were developed to predict breast cancer using different number of features. It was concluded that LR-RFE contributed to better classification performance and improved model accuracy. Therefore, we chose to use LR-RFE on genetic variation to observe the effectiveness of improving the accuracy of risk score prediction.

**Fig. 4:**
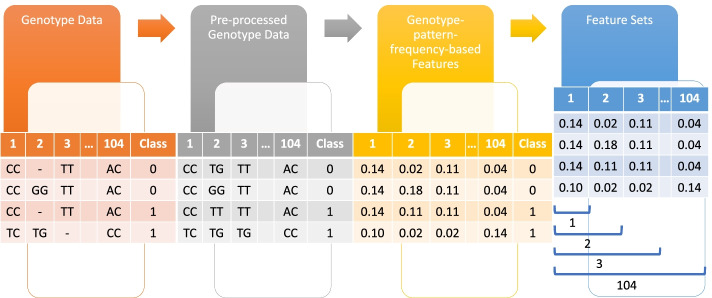
Overview of genotype-pattern-frequency-based features

**Fig. 5:**
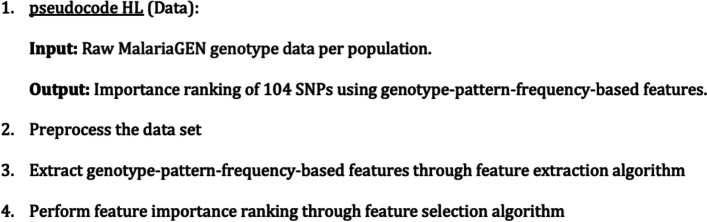
High-level pseudocode of the feature extraction and selection stage

Subsequently, to explore different sets of features for predicting malaria risk score, the results obtained are then utilized to generate 104 feature sets based on feature importance ranking. For example, the first feature set consists of the top one feature, whereas the second feature set consists of the top two features, and so on as shown in Fig. 4. Finally, the last feature set consisting of all 104 features is used to compare the efficacy of other feature sets. For each feature set, the cumulative effects of features are used as the target variable, whereby the wGRS and wGRS + GF are summed, respectively. Figure 5 shows the high-level pseudocode up to this stage.

### Model development

Preprocessed datasets from the previous stage are split into two independent datasets, namely Test-1 (15%) and Test-2 (85%). The study in [70] recommended the split percentage.

Test-1 and Test-2 are split into seven equally-sized random groups by using sevenfold cross-validation to prevent overfitting. Three machine learning models are trained, namely, Light Gradient Boosting Machine (LightGBM), eXtreme Gradient Boosting (XGBoost), and Ridge regression. We choose these machine learning algorithms because they are rarely used to analyse SNP genotype data and have been proven to have higher efficiency and faster training speed in other machine learning domains. For each machine learning model, the Test-1 dataset is utilized to obtain the best parameters through a grid search. These best parameters are then used on the Test-2 dataset for the machine learning prediction models.

### Model evaluation

#### Performance metrics

The final stage of the pipeline in Fig. 1 evaluates the performance of each model in predicting individual malaria risk scores. The outcome is a continuous risk score, so the Mean Absolute Error (MAE) and Root Mean Squared Error (RMSE) metrics are utilized [71]. The MAE measures the average residual error between the target and predicted values. On the other hand, the RMSE measures the square root of the average squared residual error between the target and predicted values. Unlike other metrics, MAE and RMSE are negatively-oriented scores, where a smaller value indicates better model performance. In other words, the lower the MAE or RMSE value, the higher the prediction accuracy.

All code was developed using the Python programming language, and simulations were performed on a machine with Apple M1 Max processor and 32 GB of memory. All methods were carried out in accordance with relevant guidelines and regulations.

## Results

### Optimization of feature importance ranking

**Fig. 6:**
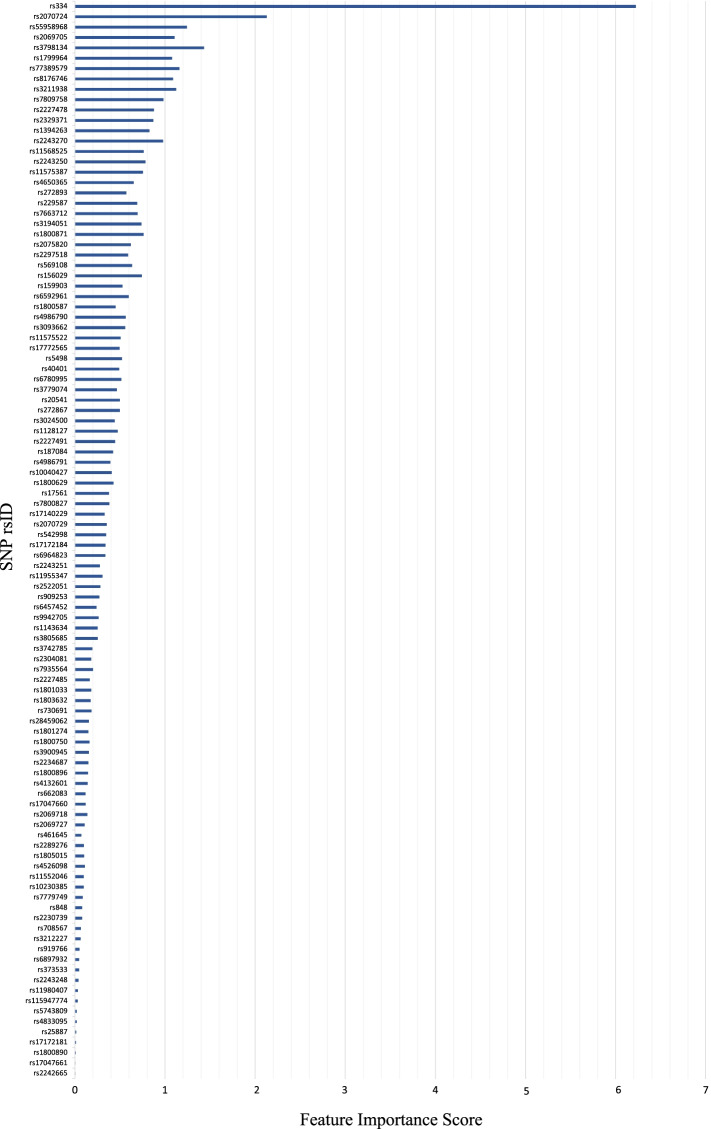
Feature importance ranking of all 104 SNPs, computed using the proposed feature extraction algorithm with LR-RFE

**Fig. 7:**
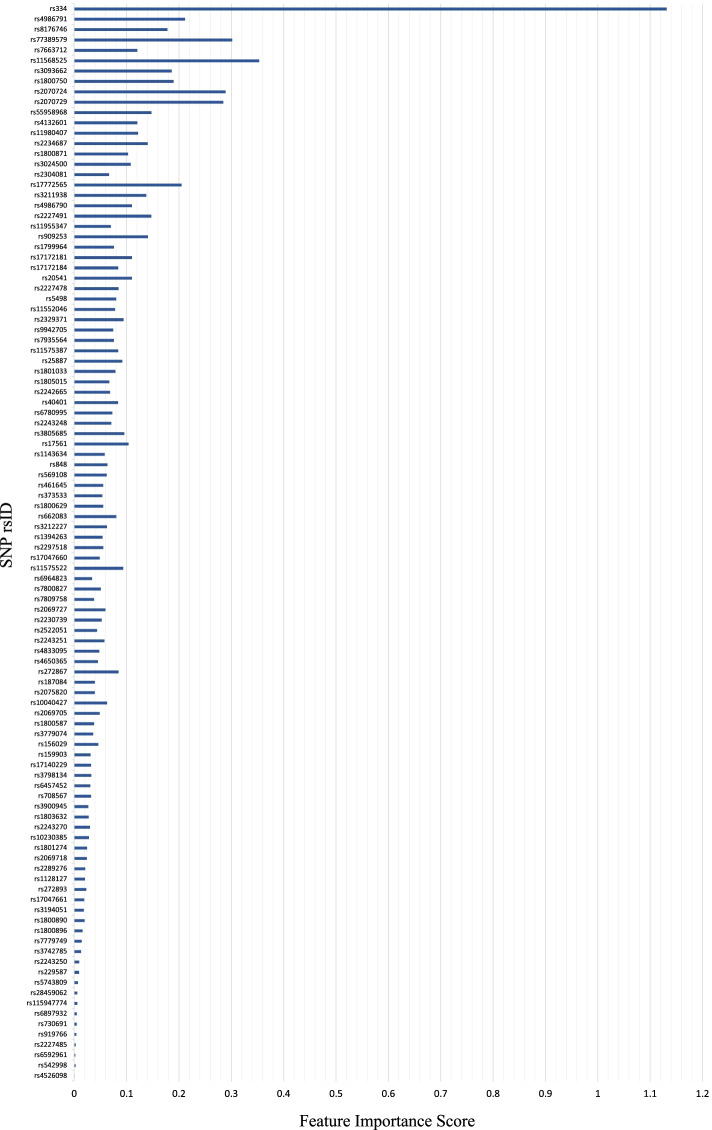
Feature importance ranking of all 104 SNPs, computed using the benchmark feature extraction algorithm with LR-RFE

Figures 6 and 7 highlight the feature importance ranking and importance scores for all 104 SNPs, starting from the most important to the least important in the context of malaria risk score prediction. For readability purposes, we only show the absolute value of the scores. The ranks in Fig. 6 are computed using the proposed feature extraction algorithm with LR-RFE as detailed in the Methods section. (We also provide a list of these features ranked in ascending order in the Additional file 2.) By contrast, Fig. 7 is based on the feature extraction algorithm that substitutes homozygous major, heterozygous, and homozygous minor genotypes with numerical values {0, 1, 2} with LR-RFE, as benchmark comparisons.

**Fig. 8:**
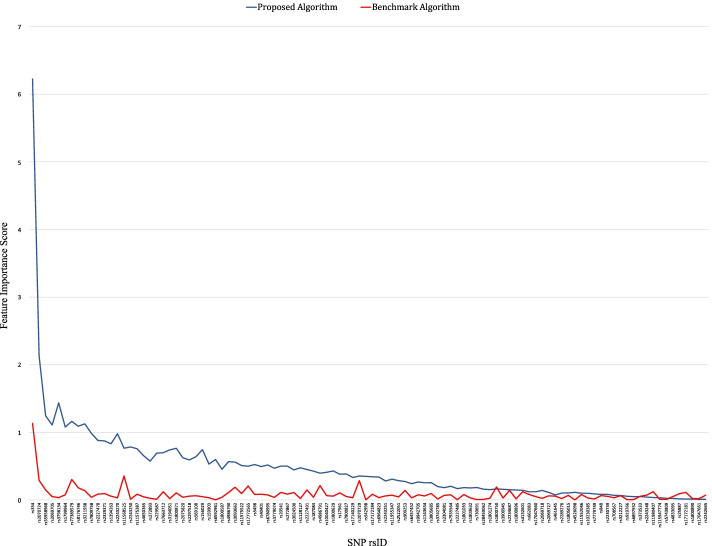
Comparison of feature importance scores using different feature extraction algorithms with LR-RFE: (1) proposed algorithm and (2) benchmark algorithm

The results of two different feature extraction algorithms with LR-RFE are summarized in Fig. 8, which shows the comparative feature importance of all 104 SNPs. These results indicate that LR-RFE ranks features and calculates their importance scores differently depending on the feature extraction algorithm in use. For example, *rs334* was ranked as the 1st feature with an importance score of 6.224 in Fig. 6. However, when comparing with the benchmark in Fig. 7, it was ranked as the 1st feature with an importance score of 1.1314 instead. The higher the score, the more prominent the feature is in predicting malaria risk score. Of note, previous findings from MalariaGEN [12, 15, 18–22] and prior studies [10, 11] have proven that the *HBB* gene is the major genetic risk factor for malaria, and *rs334* is an SNP from *HBB*. Thus, the proposed algorithm with LR-RFE can be considered a promising method, where it can identify *rs334* with a higher importance score compared to the benchmark algorithm.

### Analysis of model prediction results

We train and evaluate three machine learning regression models to predict individual risk scores towards malaria susceptibility. Results across different feature sets based on wGRS and wGRS + GF as target variables are reported in Additional files 3 and 4, respectively. These results show that reducing the feature set sizes lowers the RMSE scores in contrast to increasing the sizes, which lowers the MAE scores. This is as expected because, in RMSE, the errors are squared before being averaged, which gives higher errors more weight resulting in the metric being more sensitive to isolated outliers than MAE [71]. In other words, increasing the feature set size increases the number of outliers, which in turn increases the number of errors. Hence, MAE is a better indicator of the model's performance than the RMSE and is used in this study as the evaluation metric.

When based on wGRS alone, the best performing model is LightGBM, which achieves an MAE score of 0.0373 when trained on a single feature, i.e., *rs334*, compared to the MAE score of 1.1104 when trained on all the 104 features. In contrast, based on wGRS + GF as the target variable, the MAE scores indicate that all models performed significantly better than wGRS alone. LightGBM obtains the best performance, yielding an MAE score of 0.0033 when only a single feature, i.e., *rs334*, is utilized. Compared to wGRS, the MAE scores obtained across different feature sets based on wGRS + GF as the target variable is much lower, indicating higher accuracy.

**Fig. 9:**
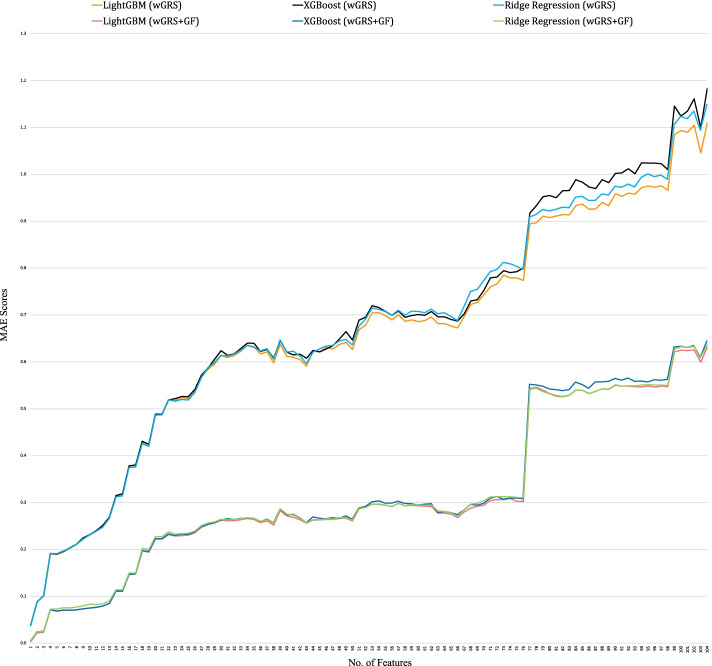
Performance analysis of the wGRS-based and wGRS + GF-based models with respect to MAE scores and feature sets

Regardless of whether wGRS or wGRS + GF are utilized, all models achieve the lowest MAE scores when trained solely on a feature, i.e., *rs334*. On the other hand, they achieve the highest MAE scores when trained with all the 104 features. Furthermore, there is a marginal difference between the scores obtained by using the default parameters and the best parameters. We also provide a graph (Fig. 9) containing the MAE scores against the number of features based on wGRS and wGRS + GF.

## Discussion

The genetic-based method of predicting the malaria risk is a powerful and feasible method that can strengthen the existing prevention strategies for malaria elimination. Traditional GWAS has achieved fruitful results in exploring the genetic risk associated with malaria through SNPs identification in specific populations [72]. GWAS is conducted to understand the disease, genes, and pathways, but it is not designed to predict whether an individual may develop the disease. On the other hand, machine learning methods can identify interacting genetic variants [61, 73] and, thus, are used in predicting complex genetic diseases, such as breast cancer [64], asthma [74], and Crohn’s disease [75].

This study puts forward a hypothesis that machine learning methods can quantify individual risk scores of susceptibility to malaria based on genetic variants. We propose a genotype-pattern-frequency-based feature extraction algorithm with LR-RFE for feature selection, where the importance of genotype patterns in malaria prediction was essentially highlighted in the Methods section. To evaluate the performance of our proposed method, we implemented the most common feature extraction algorithm with LR-RFE for feature selection as a benchmark comparison. We observe that LR-RFE ranks features and calculates their importance scores differently depending on different feature extraction algorithms. Thus, a suitable feature extraction algorithm may be an essential step to discover the most important markers in building an accurate prediction model. In particular, when using the proposed feature extraction algorithm, LR-RFE ranked *rs334* as the most contributing feature with an importance score of 6.224, while the baseline has a score of 1.1314. The importance score is crucial as the scores highlight the significant level of features contributing to malaria risk prediction that will affect the final prediction result. Therefore, the higher the score, the more prominent the feature is in predicting malaria risk.

This is an important result because previous findings from MalariaGEN [12, 15, 18–22] and prior studies [10, 11] have proven that the *HBB* gene is the major genetic risk factor for malaria, and *rs334* is an SNP from *HBB*. Hence, the proposed feature extraction algorithm with LR-RFE appears to be a promising method to extract the significant contributing risk factors to malaria.

We calculate wGRS and wGRS + GF as target variables to learn and model the relationship between SNPs. These scores enable us to understand the degree to which a genetic marker, i.e., an SNP, is associated with malaria via machine learning. This includes training and analysing three machine learning regression models: LightGBM, XGBoost, and Ridge regression, to predict the risk score of an individual’s susceptibility to malaria through genetic variants information only. Machine learning models are expected to simulate the relationship between SNP genotype data and target variables, explaining more genetic components of complex diseases such as malaria. Compared with GWAS research alone, building a model to predict malaria risk scores among different populations will be an essential advancement for disease prediction [76].

The wGRS based results indicate that LightGBM is the best performing model obtaining an MAE score of 0.0373 when trained solely on feature *rs334* in contrast to obtaining an MAE score of 1.1104 despite being trained on all 104 features. We further compare the performance of wGRS with wGRS + GF to identify the better performing model. Surprisingly, the MAE scores obtained across different feature sets for all models is much lower than solely utilizing wGRS, indicating significantly better performance. The best performing model is LightGBM, which achieves an MAE score of 0.0033 when trained solely on feature *rs334*. The wGRS + GF approach is a novelty, as to date, researchers have only used wGRS [58–60], which is insufficient for malaria risk score prediction as genotype patterns are essential in malaria prediction. It is also worth noting that when the number of features utilized to train the model increases, the data correlation becomes complex. Thus, the performance of the model decreases. Furthermore, as the model's MAE score is very low, these findings support our hypothesis that genetic variants are efficient markers of the disease and, therefore, may be used for future machine learning predictions.

We also note that both wGRS and wGRS + GF approaches achieve the best performing models when solely trained on feature *rs334*. This result is as expected, as multiple prior studies [10–12, 15, 18–22] have identified SNP *rs334* from the *HBB* gene as malaria's main genetic risk factor. However, it is insufficient to train on a single feature as recent studies have shown that each SNP is associated with disease development only to an extent, and complex interactions between features may improve the predictive ability of the model [10, 77]. Moreover, many genes are involved in the genetic basis of malaria's susceptibility or resistance, making prediction complicated on several levels [10]. For instance, even though the *rs334* feature may provide the most potent predictive power, the remaining features may improve the prediction further.

In addition, we compare the prediction performance of different feature combinations determined using the feature extraction algorithms with LR-RFE. To represent small and large feature sets respectively, we used sets 10, 20, 30, 40, 50, 60, 70, 80, 90, and 100. The results are summarized in Table 2. We note that the feature set generated using the proposed feature extraction algorithm with LR-RFE achieved slightly better MAE scores when the feature sizes were between 50 and 70. In contrast, the feature set generated using the baseline with LR-RFE obtained slightly better MAE scores when the feature sizes were between 20 and 40. However, there is only a marginal difference in the MAE scores obtained for feature sizes 10, 80, 90, and 100.

**Table 2:** Comparison of prediction performance between different feature combinations determined using the feature extraction algorithms with LR-RFE

Feature set	Proposed feature extraction algorithm			Baseline feature extraction algorithm		
	LightGBM	XGBoost	Ridge regression	LightGBM	XGBoost	Ridge regression
wGRS						
10	0.2304	0.2313	0.2313	0.2035	0.2037	0.2027
20	0.4862	0.4883	0.4868	0.2678	0.2675	0.2706
30	0.6135	0.6238	0.6141	0.3471	0.3514	0.3622
40	0.6114	0.6204	0.6209	0.4683	0.4726	0.5050
50	0.6263	0.6464	0.6362	0.8010	0.8124	0.8274
60	0.6856	0.7009	0.7078	0.8145	0.8330	0.8491
70	0.7431	0.7522	0.7731	0.8129	0.8321	0.8260
80	0.9077	0.9546	0.9219	0.9047	0.9509	0.9394
90	0.9586	1.0018	0.9744	0.9302	0.9704	0.9538
100	1.0934	1.1240	1.1236	1.0318	1.1065	1.0641
wGRS + GF						
10	0.0748	0.0749	0.0831	0.0584	0.0597	0.0600
20	0.2220	0.2231	0.2274	0.0747	0.0751	0.0760
30	0.2625	0.2624	0.2641	0.1265	0.1285	0.1352
40	0.2713	0.2736	0.2751	0.1743	0.1800	0.1931
50	0.2605	0.2644	0.2625	0.5646	0.5656	0.5671
60	0.2929	0.2945	0.2944	0.5660	0.5678	0.5721
70	0.2940	0.2981	0.3034	0.5354	0.5345	0.5359
80	0.5323	0.5421	0.5317	0.5602	0.5610	0.5670
90	0.5501	0.5646	0.5509	0.5437	0.5463	0.5488
100	0.6249	0.6333	0.6323	0.5929	0.6041	0.6003

Our findings further reaffirm that prediction performance is affected by the complex interactions between the susceptibility or resistance features (i.e., SNP) in predicting malaria risk. This is because each SNP is not equally important due to its susceptibility or resistance levels. Therefore, when combined, the impact of the SNPs interactions differs from one feature set to another. Hence, we hardly justify which feature combination is the most important for malaria prediction as each feature has a particular contribution to malaria development.

Extremely statistically significant differences in MAE scores, i.e., *p*-value < 0.0001, are obtained using wGRS and wGRS + GF via one-way Analysis of Variance (ANOVA) test. Of note, we performed a normality test for the data distribution to ensure no violation of the one-way ANOVA test's normality assumption. We used the skewness and kurtosis index to identify the normality of the data, obtaining the range of [-0.4204, + 0.4005] for skewness and [-0.8025, -0.2566] for kurtosis. The data is considered normal if the skewness falls in the range of [‐2, + 2] [78, 79] and the kurtosis falls in the range of [‐7, + 7] [78, 79]. Hence, we can conclude the MAE scores of the models are normally distributed. To further compare the two methods, we also provide Table 3 summarizing the *p*-values obtained with the best parameters and Table 4 summarizing the *p*-values obtained with the default parameters.

**Table 3:** P-values obtained with best parameters

LightGBM	XGBoost	Ridge regression
2.52E-24	8.56E-24	2.13E-24

Compares the *p*-values of MAE scores using the aforementioned risk scores with best parameters

**Table 4:** *P*-values obtained with default parameters

LightGBM	XGBoost	Ridge regression
6.41E-24	7.76E-23	2.59E-23

Compares the *p*-values of MAE scores using the aforementioned risk scores with default parameters

This study has obtained promising results in predicting the risk score of individual susceptibility to malaria. We believe that our findings hold great promise for individual malaria risk score prediction and contribute in bridging the implementation gap between healthcare practitioners and computer scientists. The pipeline of the model’s development emphasized in this study can fully be reproduced and thus, be used as a base towards model retraining with new data.

Our study, based on 20,817 individuals from eleven populations, provides a basis for further exploration and improvement of the machine learning models, where individuals from different continents can be included. In addition, to explore more comprehensive solutions and strengthen our proposed method, alternate hypotheses may be considered, namely, developing a machine learning model that integrates blood group and SNP genotype data of malaria-affected individuals to characterize the genetic component and complexity of the disease. There is substantial evidence showing that blood group A is very susceptible to malaria, whereas blood group O can prevent malaria [80, 81].

## Conclusions

We developed machine learning-based prediction models which utilized SNPs genotype data and calculated wGRS and wGRS + GF as target variables to quantify the risk score of an individual’s susceptibility to malaria. More precisely, we employed an approach that consists of the proposed genotype-pattern-frequency-based feature extraction algorithm with LR-RFE to identify the SNPs’ significant level implication to malaria. Results show that this approach identified and ranked SNP *rs334* (a major genetic risk factor for malaria proved by previous studies) as the most contributing feature with an importance score of 6.224 compared to the baseline which only yields an importance score of 1.1314. (This suggests that our approach has the potential to discover significant genetic markers for other diseases as well.) Furthermore, LightGBM, a tree-based model, is the best-performing model in this study. It is also found that compared with wGRS alone, the model trained based on SNP genotype data and wGRS + GF obtains a lower MAE scores, and it is also a novelty as far as the literature on risk scores is concerned. To a larger extent, we have shown a promising method that demonstrates how machine learning can augment the insights derived from GWAS to quantify an individual’s risk score for a particular disease.

## Supplementary Information

**Additional file 1.** **Table S1.** General information on the 104 SNPs used in this study.

**Additional file 2.** **Table S2.** A list of features ranked in ascending order obtained from our approach.

**Additional file 3.** **Table S3.** Results of different feature sets based on SNPs genotype data with wGRS as the target variable. Performance metrics are based on MAE and RMSE scores.

**Additional file 4.** **Table S4.** Results of different feature sets based on SNPs genotype data with wGRS+GF as the target variable. Performance metrics are based on MAE and RMSE scores.

## Data Availability

The datasets analysed during the current study are available in the MalariaGEN Consortial Project 1 (https://www.malariagen.net/data/genome-wide-study-resistance-severe-malaria-eleven-populations-version-2); EGAD00010001799, EGAD00010001738, EGAD00010001818, EGAD00010001737, EGAD00010001739, EGAD00010001734, EGAD00010001736, EGAD00010001740, EGAD00010001743, EGAD00010001741, EGAD00010001742, EGAD00010001733, EGAD00010001735.

## Abbreviations

ANOVA: Analysis of variance
GWAS: Genome-wide association study
HBB: Hemoglobin subunit beta
LightGBM: Light gradient boosting machine
LR: Logistic regression
LR-RFE: Logistic regression and recursive feature elimination
MAE: Mean absolute error
MalariaGEN: Malaria genomic epidemiology network
RFE: Recursive feature elimination
RMSE: Root mean squared error
SNP: Single nucleotide polymorphism
wGRS: Weighted genetic risk score
wGRS + GF: Weighted genetic risk score with genotype frequency
XGBoost: EXtreme gradient boosting
